# Nrf2 Activation and Antioxidant Properties of Chromone-Containing MTDLs for Alzheimer’s Disease Treatment

**DOI:** 10.3390/molecules30092048

**Published:** 2025-05-04

**Authors:** Alexey Simakov, Stecy Chhor, Lhassane Ismaili, Hélène Martin

**Affiliations:** 1 Université Marie et Louis Pasteur, INSERM UMR1322 LINC, F-25000 Besançon, France; 2 Université Marie et Louis Pasteur, EFS, INSERM UMR1098 RIGHT, F-25000 Besançon, France

**Keywords:** antioxidant effect, Nrf2, chromone, MTDLs, Alzheimer’s disease

## Abstract

Alzheimer’s disease (AD) is a devastating neurodegenerative disorder affecting millions worldwide and imposing a significant social and economic burden. Despite extensive research, there is still no effective cure for this disease. AD is multifactorial and involves multiple etiopathogenic mechanisms, one of which is oxidative stress. Consequently, the Nrf2/ARE pathway, which regulates the expression of cellular defense genes, including those for antioxidant enzymes, is considered to be a prospective therapeutic target for AD. Meanwhile, multitarget-directed ligands (MTDLs) are a promising approach for developing effective AD medications. In this regard, we evaluated the antioxidant potential of eight chromone-containing MTDLs in vitro, including Nrf2 transcriptional activation potencies, Nrf2/ARE downstream genes activation, and antioxidant effects in vitro. All tested compounds effectively activated the Nrf2/ARE pathway. Notably, compounds **4b**, **4c**, **4f**, and **4h** demonstrated the highest Nrf2 activation potencies, while compounds **4b**, **4c**, **4d**, and **4g** significantly induced the expression of Nrf2-target antioxidant genes, specifically NQO1 and HO1. Additionally, compound **4d** exhibited a significant antioxidant effect in vitro. These findings encourage further investigation of the studied compounds, with particular emphasis on compound **4d** as the most promising candidate.

## 1. Introduction

Alzheimer’s disease (AD) is a devastating and irreversible neurodegenerative disorder characterized by the progressive loss of neurons, leading to cognitive impairment, particularly dementia [1]. AD primarily affects the elderly and is the most common cause of dementia in this population [2]. With global population aging, the number of dementia cases is estimated to reach 152 million by 2050 [2,3]. Along with the incidence of AD, the social and economic burden of the disease is expected to increase significantly [4]. To date, there is no effective cure for AD, and available medications have a low impact on the disease progression [5]. In addition, newly developed drugs tend to fail during the phase I and II of clinical trials [6].

The evidence accumulated suggests that AD involves multiple interconnected etiopathogenic mechanisms including *β*-amyloid (A*β*) assemblies [7], tau-protein aggregation and hyperphosphorylation [8], low levels of acetylcholine [9], mitochondrial dysfunction [10], altered homeostasis of biometals [11], neuroinflammation [12], and oxidative stress [13,14,15].

Oxidative stress is defined as an imbalance between pro-oxidants and antioxidants, leading to the disruption of redox homeostasis and macromolecular damage [16]. It is associated with increased production of reactive oxygen species (ROS) and reactive nitrogen species (RNS) in cells. In recent years, oxidative stress has received greater attention for its role in the development of AD [15]. There is evidence that oxidative damage occurs during the early stages of AD, even before the appearance of main biomarkers such as A*β* plaques and tau tangles [17]. Oxidative stress appears to act as a link between different pathological features of AD. Mitochondrial dysfunction is considered a primary source of ROS, as mitochondria are the major producers of ROS during oxidative phosphorylation [15]. ROS-induced mitochondrial damage further increases ROS production, triggering a cascade of mitochondrial dysfunction that disrupts synaptic activity [18]. Additionally, biometal imbalances typical for AD catalyze ROS production [19]. ROS contribute to A*β* aggregation and plaque formation, as well as tau hyperphosphorylation and neurofibrillary tangle (NFT) formation, leading to impaired synaptic transmission, neurotoxicity, and neuronal death over time [20]. Notably, A*β* plaques and NFTs amplify ROS production, creating a vicious cycle [14]. ROS also activate microglia and astrocytes, which release pro-inflammatory cytokines that intensify neuronal damage [13].

A crucial mechanism regulating the activation of the antioxidant response is the Nrf2/ARE pathway. Nuclear factor erythroid-2-related factor 2 (Nrf2) is a transcription factor that, under non-stressed conditions, is located in the cytosol in a complex with Kelch-like ECH-associated protein 1 suppressor (Keap1) and Cullin3 (Cul3) proteins. It is subsequently degraded through ubiquitination and proteasomal degradation. However, under oxidative stress conditions, cysteine residues in the Keap1 structure undergo oxidation, inducing a conformational change that leads to the dissociation of the Nrf2-Keap1-Cul3 complex. As a result, free Nrf2 translocates to the nucleus, where it binds to the antioxidant response element (ARE) in the promoter regions of its target genes. These genes encode antioxidant and detoxifying enzymes, such as NAD(P)H:quinone dehydrogenase 1 (NQO1), heme oxygenase-1 (HO-1), glutathione S-transferase (GST), as well as glutamate-cysteine ligase catalytic subunit (GCLC) and glutamate-cysteine ligase regulatory subunit (GCLM), which are involved in glutathione synthesis [21].

Given the complex etiopathology of AD, it has become evident that employing a combined approach targeting multiple molecular targets of AD may lead to greater therapeutic success [22]. Consequently, researchers are paying more attention to the multi-target-directed ligands (MTDLs), simultaneously modulating multiple cellular pathways associated with AD pathology [23].

**Figure 1:**
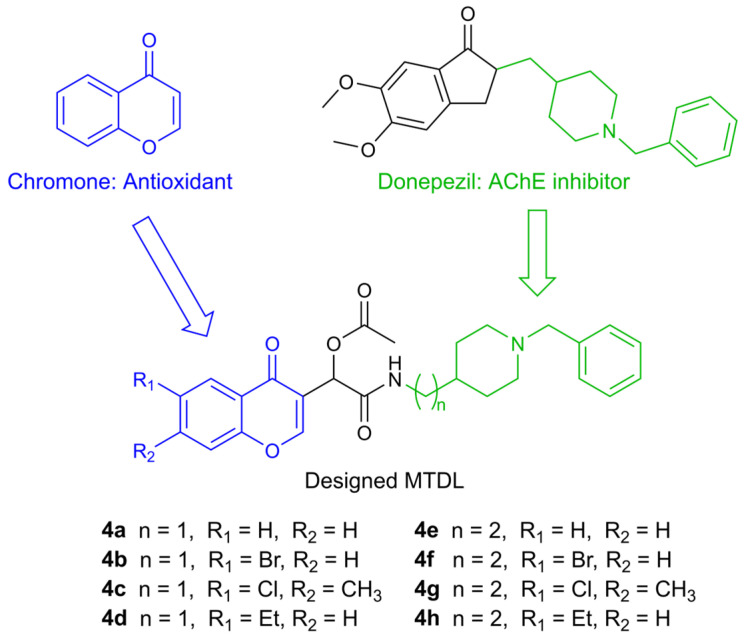
Design of racemic chromone–donepezil hybrids.

In accordance with this approach, several MTDLs were reported by Malek et al. in 2019 [24]. These compounds consist of a chromone moiety, which is responsible for the antioxidant effect, and a pharmacophore derived from donepezil, one of the first approved drugs for AD, which acts as an acetylcholinesterase inhibitor (AChEI) (Figure 1).

Based on previous results demonstrating the promising antioxidant effects of the reported compounds, we further investigated the antioxidative potential of these MTDLs in vitro and hypothesized their ability to induce the Nrf2/ARE pathway, according to previous reports on structurally similar molecules containing a chromone group [25,26,27,28,29].

## 2. Results

### 2.1. Nrf2 Transcriptional Activation Potencies

The NRF2/ARE-luciferase reporter HEK293 stable cell line was exposed to non-cytotoxic concentrations of each compound, determined in preliminary experiments (Table 1). Indeed, all tested compounds demonstrated no cytotoxic effects at concentrations up to 10 µM, except for **4b** and **4c**, which exhibited significant cytotoxicity starting at 10 µM on this cell line.

**Figure 2:**
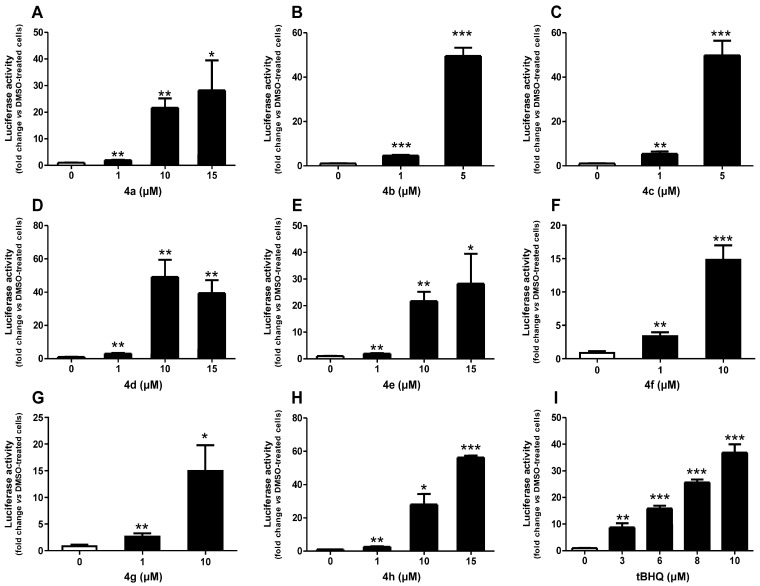
Effect of compounds **4a** (**A**), **4b** (**B**), **4c** (**C**), **4d** (**D**), **4e** (**E**), **4f** (**F**), **4g** (**G**), **4h** (**H**), and a reference compound tBHQ (**I**) on Nrf2/ARE/luciferase activity after 24 h of treatment. Data are presented as the mean ± SEM of three independent experiments. * *p* ≤ 0.05, ** *p* ≤ 0.01, and *** *p* ≤ 0.001 with respect to control cells (one-way ANOVA with post hoc Dunnett’s test).

Figure 2 illustrates the effect of the tested compounds at non-cytotoxic concentrations on the Nrf2/ARE pathway activation. As expected, the reference compound tBHQ, a well-known activator of the Nrf2/ARE pathway [30], led to a concentration-dependent activation, reaching a 37-fold increase at 10 µM, which was the highest concentration tested. Notably, all studied compounds induced activation of the Nrf2/ARE pathway, including concentrations as low as 1 µM. Compound **4h** showed maximum induction at 15 µM, with just under a 60-fold increase. Similarly, 15 µM of **4a** and **4e** induced the Nrf2/ARE pathway by approximately 30-fold. Both **4f** and **4g** at 10 µM induced the pathway by 15-fold. A significant induction of more than 40-fold was observed at 10 µM of **4d**, exceeding the induction of tBHQ at the same concentration. Interestingly, compounds **4b** and **4c**, tested at lower concentrations due to their cytotoxicity, demonstrated an approximately 50-fold induction at 5 µM, which was also higher than that of tBHQ.

Additionally, CD values, which correspond to the concentrations required to double the specific reporter activity, were calculated for these compounds to assess their relative potencies and are presented in Table 2. The CD values of both **4d** and **4g** were 0.7 µM compared to 0.6 µM for the reference compound tBHQ. Moreover, **4b**, **4c**, **4f**, and **4h** exhibited greater activity than tBHQ, with CD values of 0.4, 0.3, 0.4, and 0.5 µM, respectively. In contrast, **4a** and **4e** had CD values of 1.0 µM, making them approximately 1.7 times less potent than tBHQ.

### 2.2. Nrf2 Downstream Gene Activation

Preliminary cell viability assay was performed in the SH-SY5Y cell line after 24 h of treatment (Table 3). These experiments demonstrated that **4b**, **4c**, and **4g** were toxic at 10 µM in the SH-SY5Y cell line. Therefore, these concentrations of the compounds were excluded from subsequent assays.

**Figure 3:**
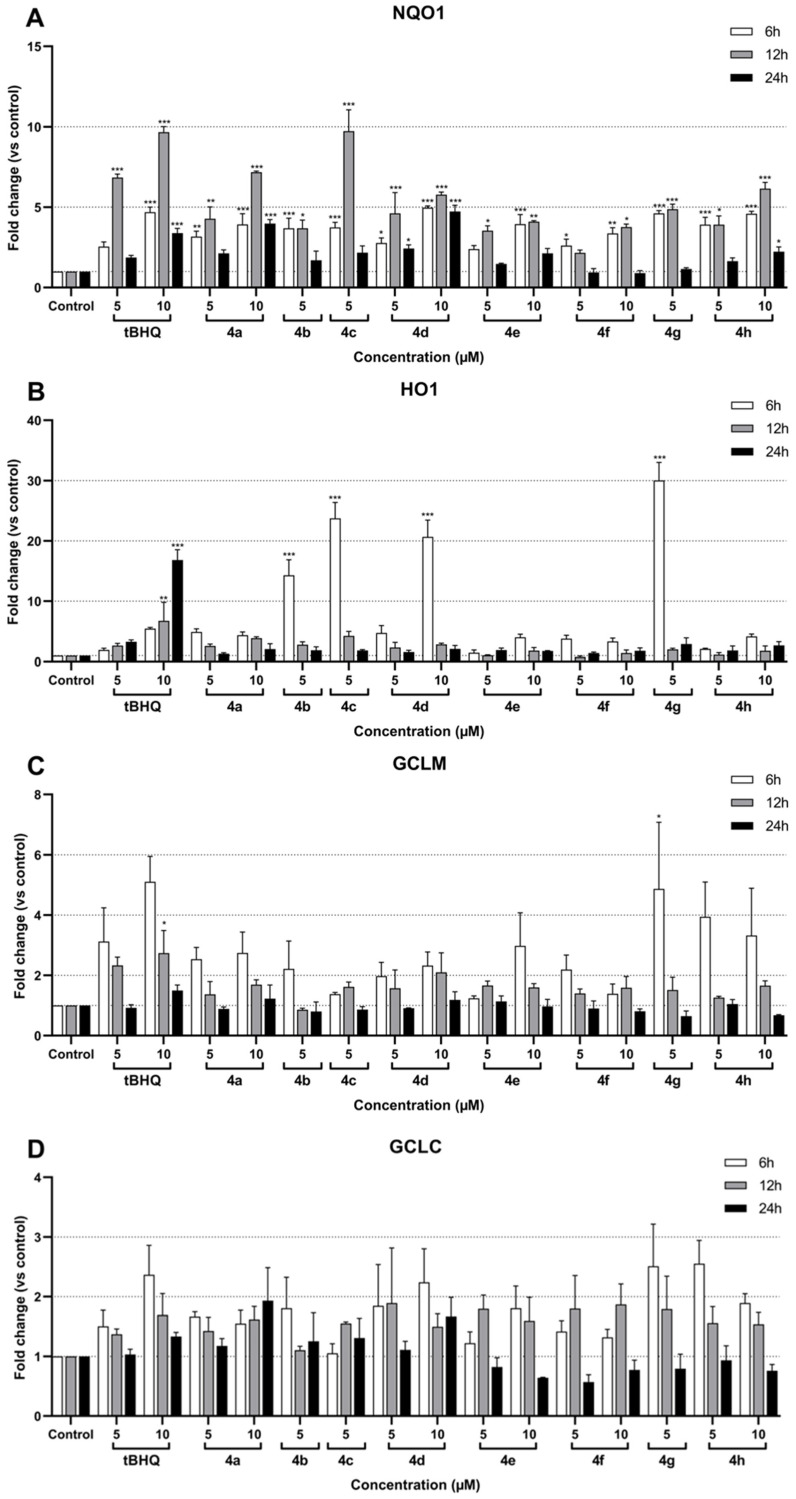
Effect of compounds **4a**–**4h** on NQO1 (**A**), HO1 (**B**), GCLM (**C**), and GCLC (**D**) mRNA expression in SH-SY5Y cells after 6 h, 12 h, and 24 h of treatment. Data are presented as the mean ± SEM of three independent experiments. * *p* ≤ 0.05, ** *p* ≤ 0.01, and *** *p* ≤ 0.001 with respect to control cells (one-way ANOVA with post hoc Dunnett’s test).

The results for the remaining conditions are presented in Figure 3.

RT-qPCR analysis was performed after 6 h, 12 h, and 24 h of treatment with the different compounds. The corresponding data showed a significant induction of NQO1 in treated SH-SY5Y cells compared to the control (Figure 3A). The NQO1 induction ranged from 1- to 10-fold depending on the compound and the time of treatment. The greatest increases were observed after 12 h of treatment, whereas results after 24 h were not significant for most compounds. After 6 and 12 h of treatment, all tested compounds successfully exhibited an induction of NQO1 expression, rising above 2-fold. Among these, compounds **4a**, **4d**, and **4h** at 10 µM and compounds **4c** and **4g** at 5 µM demonstrated at least 5-fold upregulation of NQO1 after 12 h of treatment. Interestingly, in contrast to other compounds, 10 µM **4d** showed consistent NQO1 expression at 6, 12, and 24 h, fluctuating around 5-fold. Similarly, the reference compound tBHQ induced NQO1 expression by more than 5-fold; specifically, it showed a 7-fold induction at 5 µM and just under 10-fold at 10 µM after 12 h of treatment. The most significant responses were observed after 12 h with **4a** at 10 µM, which showed an approximately 7-fold induction, and with **4c** at 5 µM, which peaked at around 10-fold induction. These responses were comparable to the upregulation observed with 5 µM and 10 µM tBHQ, respectively, at the same time point.

HO1 expression was significantly induced by tBHQ and the compounds **4b**, **4c**, **4d**, and **4g** (Figure 3B). Moreover, the peak induction times differed between the compounds and tBHQ, 6 h and 24 h, respectively. Interestingly, the compounds exhibited a drastic increase in expression: 5 µM **4g** induced a 30-fold increase, 5 µM **4b** induced a 15-fold increase, 5 µM **4c** induced approximately a 25-fold increase, and 10 µM **4d** induced a 20-fold increase. In comparison, 10 µM tBHQ resulted in an approximately 16-fold induction.

Although GCLM expression showed a minimal response to most compounds, the peak induction was observed after 6 h of treatment, including with tBHQ (Figure 3C). Only 10 µM tBHQ and 5 µM of compound **4g** after 6 h caused significant inductions of approximately 3-fold and 5-fold, respectively. Interestingly, compound 4 h at both 5 µM and 10 µM demonstrated pronounced inductions of around 4-fold and 3.5-fold, respectively, although these results were not statistically significant. Conversely, GCLC did not exhibit any significant induction, regardless of the compound tested (Figure 3D).

Overall, the data indicate that several test compounds, particularly **4a**, **4c**, **4d**, and **4g**, exhibit significant activation of the NQO1 and HO1 pathways, comparable to or exceeding the reference compound tBHQ, while the effects on GCLC and GCLM expression were weak and mostly insignificant.

For the subsequent assay, compounds **4e** and **4f** were excluded as the least promising candidates.

### 2.3. Antioxidant Assay In Vitro

**Figure 4:**
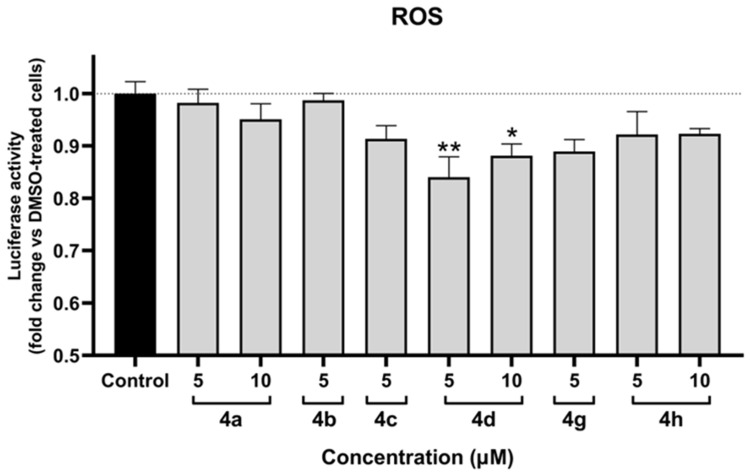
Protective effect against oxidative stress of compounds **4a**, **4b**, **4c**, **4d**, **4g**, and **4h** in SH-SY5Y cells after 12 h of treatment. Data are presented as the mean ± SEM of three independent experiments. * *p* ≤ 0.05 and ** *p* ≤ 0.01 with respect to control cells (one-way ANOVA with post hoc Dunnett’s test).

The antioxidant assay was performed in SH-SY5Y cells following 12 h of treatment. The assay was validated using the reference compound H_2_O_2_, 500 µM of which induced a 2-fold increase in ROS levels. As illustrated in Figure 4, only **4d** exhibited statistically significant antioxidant effects. Notably, both tested concentrations of **4d** demonstrated an effect, with 5 µM showing a stronger response than 10 µM. Additionally, **4g** showed a tendency towards antioxidant activity; however, the results were not statistically significant.

## 3. Discussion

This study aimed to investigate whether the set of chromone-containing MTDLs is able to activate the Nrf2/ARE pathway and provide an antioxidant effect in vitro.

Our assumption is based on the fact that chromones contain an *α*,*β*-unsaturated ketone group, which exhibits electrophilic properties and is also known as a Michael acceptor [31]. The canonical mechanism of Nrf2/ARE activation involves the oxidation of cysteine residues in the Keap1 structure, which subsequently disrupts the Nrf2-Keap1-Cul3 complex and eventually leads to transcription of the genes of antioxidant and cytoprotective enzymes [21]. The cysteine residues are nucleophiles and can play the role of Michael donors. Therefore, oxidation of Keap1 cysteine residues via a Michael addition reaction with *α*,*β*-unsaturated ketones is considered the mechanism through which chromones activate the Nrf2/ARE pathway [32].

In addition, there is evidence of alternative mechanisms of Nrf2 activation by chromone-containing structures, including Nrf2 phosphorylation by various kinases, leading to its nuclear translocation, dissociation of the Nrf2-Keap1-Cul3 complex via phosphorylation and Keap1 autophagic degradation, decreased Nrf2 ubiquitination, and epigenetic alterations of Nrf2 [33]. Further investigations are necessary to understand the exact mechanism of Nrf2 activation by the tested molecules.

Based on the CD values associated with Nrf2 transcriptional activation, the length of the chain (n = 1 or 2) separating the amide group from the benzylpiperidine does not appear to play a determining role in the activity, regardless of the nature of the substituents on the aromatic ring. Indeed, compounds **4a** and **4e** display similar CD values, as do compounds **4b** and **4f**. Slight variations are, however, observed for the **4c**/**4g** and **4d**/**4h** pairs.

For a given chain length, the presence of either an electron-donating or electron-withdrawing group on the aromatic ring generally enhances activity compared to their unsubstituted analogues. Compounds **4a** and **4e**, which lack substituents, are thus the least active in their respective series.

More interestingly, compounds bearing an electron-withdrawing group such as bromine are consistently more active than their counterparts substituted with an electron-donating group such as ethyl.

Finally, the simultaneous presence of both an electron-withdrawing group (chlorine) and an electron-donating group (methyl) leads to contrasting effects: when n = 1, the corresponding compound is the most active among all substituted derivatives, whereas when n = 2, it becomes the least active of the same group.

Additionally, the toxic effects of **4b** and **4c** in SH-SY5Y and HEK cells correlate with the presence of a halogen group in their structure, as well as with their activity compared to the other compounds. In addition, **4f** and **4g**, which are halogen-containing molecules, appear to be less toxic; however they did exhibit toxicity starting at 15 µM in HEK cells, and **4g** also demonstrated toxic effects at 10 µM in SH-SY5Y cells.

Regarding Nrf2/ARE downstream genes, their time-dependent induction could be due to several limiting factors, including promoter accessibility [34,35] and transcriptional co-activators’ availability [21,35]. Moreover, target genes can launch both positive feedback loops, increasing Nrf2 translocation over time, and negative feedback loops, downregulating pathway activation [36]. In addition, temporal difference in gene induction depends on the compound itself, its metabolic stability, rate of degradation, and cellular retention.

NQO1 showed a more consistent overall induction compared to other targets. In general, molecules with n = 1 induced NQO1 more strongly than their counterparts with n = 2, except for the **4d**/**4h** pair. In this pair, the effect after 12 h was comparable; however, the molecule **4d** demonstrated higher induction after 24 h of treatment, suggesting prolonged activation. This may be due to its longer retention in the cytosol or its Nrf2 stabilization via phosphorylation [37]. Nevertheless, the highest induction was observed with the chlorine-containing compound **4c**.

NQO1 is well known for its strong and consistent activation of Nrf2/ARE-dependent transcription, whereas the induction of other targets may vary. In our study, HO-1 induction was significant only in response to the halogen-containing compounds, **4b**, **4c**, and **4g**, as well as to **4d**, which contains an electron-donating C_2_H_5_-group. Conversely, no significant induction was observed in GCLC or GCLM, except for **4g**, which showed induction in GCLM. This difference in induction across the target genes may be explained by the ability of cells to distinguish between different extracellular signaling molecules and their concentrations by activating distinct transcription factors [38].

In case of HO-1, one such factor is activating transcription factor 4 (ATF4), which regulates HO-1 transcription in addition to Nrf2 regulation [39]. ATF4 can form dimers with Nrf2 and bind to ARE as an Nrf2-ATF4 dimer [40]. Therefore, the induction of HO-1 by **4b**, **4c**, **4d**, and **4g** at their respective concentrations may be regulated not only through Nrf2 but also via ATF4, resulting in a more pronounced response [41]. Additionally, HO-1 has a distinct mechanism of ARE activation, involving the Brahma-related gene 1 (BRG1) protein [42], which may contribute to the observed differences in induction between NQO1 and HO-1.

Some discrepancies occurred between our results of the Nrf2 activation via the luciferase assay and the Nrf2 downstream gene induction, in accordance with another publication reporting flavones [29]. In the referenced article, apigenin and luteolin have induced ARE-luciferase activity in HepG2-C8 cells after 6 h and 12 h. Apigenin at 6.25 µM led to a 5.5-fold induction after 12 h, while the same concentration of luteolin showed an almost 4-fold induction at the same time point. However, substantial differences have not been observed in NQO1 and HO-1 gene expression alteration by these flavones.

In a previous study, oxygen radical absorbance capacity (ORAC) assays demonstrated that the highest antioxidative power was observed in the tested compounds in the following descending order: **4e**, **4b**, **4g**, and **4d** [24]. However, in in vitro settings, a significant antioxidant effect was detected only for **4d**, suggesting that within cells, the intrinsic antioxidant properties of these compounds, which stem from their chemical structures, are not sufficient to produce a detectable, significant antioxidant effect. This indicates that the Nrf2/ARE-related antioxidant response of the tested compounds prevails over their chemical structure-based antioxidant activity in vitro.

It is also notable that both tested concentrations of **4d** exhibited an effect, with 5 µM producing a stronger response than 10 µM. This finding suggests that higher concentrations of **4d** may induce oxidative stress to an extent that outweighs its antioxidant effect.

## 4. Materials and Methods

### 4.1. Compounds Preparation

The tested MTDL compounds were synthesized using sustainable chemistry methods, specifically the “one-pot” Passerini reaction. All compounds showed at least 95% purity [24] see Supplementary Materials. Detailed information on the synthesis process is available in the previous report [24]. Afterwards, compounds **4a**–**4h** were dissolved in 100% dimethyl sulfoxide (DMSO) and diluted with a culture medium to obtain the final test concentrations.

### 4.2. Nrf2 Transcriptional Activation

The NRF2/ARE-luciferase reporter HEK293 stable cell line (Signosis, Santa Clara, CA, USA) was maintained in high-glucose Dulbecco’s MEM (DMEM-F12) medium (Dutscher, Bernolsheim, France) supplemented with 10% fetal bovine serum (FBS) and 1% penicillin–streptomycin at 37 °C in a 5% CO_2_ humidified atmosphere. The cells were seeded at a density of 2 × 10^4^ cells per well in the same medium in white 96-well microtiter plates. After 48 h of incubation, the culture medium was replaced with fresh DMEM-F12 medium supplemented with 0.1% FBS and containing various concentrations of the test compounds, tert-butylhydroquinone (tBHQ) as a positive control, or 0.1% DMSO as a vehicle control. After 24 h of treatment, luciferase activity was measured using the Bright-Glo luciferase assay system (Promega, Charbonnières-les-Bains, France) according to the manufacturer’s instructions on a microplate reader (FLUOstar Omega, BMG LABTECH, Champigny-sur-Marne, France). All conditions were conducted in duplicate and repeated in at least three different cell passages. Luciferase activity was calculated as a fold change relative to the untreated control, which was set at 1.

To assess cell viability, the NRF2/ARE-luciferase reporter HEK293 stable cells were seeded at a density of 2 × 10^4^ cells per well in transparent 96-well plates as described above. After 48 h of incubation, the cells were treated as previously described for the luciferase activity determination. After 24 h of treatment, the medium was aspirated, and the cells were incubated with 100 µL of MTT reagent (5 mg/mL in PBS) for 2 h at 37 °C. The supernatant was removed, and 100 µL of DMSO was added to each well to dissolve the formazan. Absorbance was measured at a wavelength of 570 nm using the microplate reader. All conditions were conducted in duplicate and repeated in at least three different cell passages. Cell viability was calculated as a percentage relative to the untreated control, which was set at 100%.

### 4.3. RT-qPCR Analysis

The SH-SY5Y cell line (ATCC, Manassas, VA, USA) was cultured in DMEM-F12 (1:1) medium supplemented with 10% FBS, 1% non-essential amino acids, and 1% penicillin–streptomycin at 37 °C in a 5% CO_2_ humidified atmosphere. The cells were seeded at a density of 6 × 10^5^ cells per well in the same medium in transparent 24-well plates. After 72 h, the cells were treated with different concentrations of the test compounds or tBHQ as a positive control in the same medium used for seeding, except that 10% FBS was replaced with 0.1% FBS. Treatment was stopped after 6, 12, or 24 h by adding TRIzol reagent (Life Technologies, Saint-Aubin, France) for RNA extraction. All samples were prepared in duplicate and pooled for subsequent RT-qPCR analysis. The experiment was repeated in three independent cell passages. Total RNA was extracted using the TRIzol reagent according to the manufacturer’s instructions. cDNA was synthesized from 1 µg of total RNA with the iScript cDNA Synthesis Kit (Bio-Rad Laboratories, Marnes-la-Coquette, France) at 42 °C, following the manufacturer’s protocol. cDNA samples were then diluted in water (1:5), and 5 µL of each sample in duplicate was used for qPCR using the iQ SYBR Green Supermix Kit (Bio-Rad Laboratories, Marnes-la-Coquette, France) on the MyIQ PCR Detection System (Bio-Rad Laboratories, Marnes-la-Coquette, France). RNAse-free water and no-RT samples were included as negative controls. The primer sequences used for amplification are provided in Table 4. Gene expression was normalized to the levels of *β*-actin and GAPDH as reference genes. PCR conditions consisted of a denaturation step at 95 °C for 2 min and 45 PCR cycles (at 95 °C for 3 s and at 60 °C for 30 s). The identity and purity of the amplified products were verified by melting curve analysis at the end of each PCR assay. Normalized and averaged fluorescence ratios of the target genes were used to calculate fold changes in expression from different passages.

For cell viability assessment, the SH-SY5Y cells were seeded at a density of 1.2 × 10^5^ cells per well in fresh DMEM-F12 medium in transparent 96-well plates. After 48 h of incubation, the culture medium was replaced with fresh DMEM-F12 medium supplemented with 0.1% FBS and containing different concentrations of the test compounds or 0.1% DMSO as a vehicle control. After 24 h of treatment, MTT reagent (10 mg/mL in DMEM-F12) was added directly to each well at a final concentration of 1 mg/mL, and the plate was incubated for 2 h at 37 °C. Subsequently, 200 µL of DMSO was added to each well to dissolve the formazan crystals. Absorbance was measured at a wavelength of 570 nm using the microplate reader. All conditions were performed in triplicate and repeated in at least three independent cell passages. Cell viability was calculated as a percentage relative to the untreated control, which was set at 100%.

### 4.4. Antioxidant Assay

SH-SY5Y cells were seeded and treated with various concentrations of the test compounds or 0.1% DMSO as a vehicle control, as previously described for cell viability assessment. ROS levels were measured after a total of 12 h of treatment using the ROS Glo™ H_2_O_2_ Assay (Promega, Charbonnières-les-Bains, France) following the manufacturer’s protocol. Luminescence was measured using the microplate reader. All conditions were conducted in duplicate and repeated in at least three independent cell passages. Luciferase activity was calculated as a fold change relative to the untreated control, which was set at 1.

### 4.5. Statistical Analysis

Statistical analysis of the differences was performed with one-way ANOVA followed by post hoc Dunnett’s test, using MatLab R2022b (MathWorks, Natick, MA, USA) and Prism 8.0.1 software (GraphPad Software, San Diego, CA, USA). A *p*-value of ≤0.05 was considered statistically significant.

## 5. Conclusions

In this study, we have described the Nrf2/ARE pathway activation of eight MTDL compounds containing a chromone structure, evaluating their Nrf2 activation potency, induction of Nrf2 target genes, and antioxidant activity in vitro. Among these compounds, **4d** was identified as the most promising in terms of antioxidant effects. Furthermore, **4d** exhibited CD values for Nrf2 activation potency comparable to that of tBHQ, induced NQO1 by 5-fold, and was one of the four tested compounds capable of significantly inducing HO-1, reaching 20-fold. Additionally, it demonstrated an antioxidant effect in vitro while showing no toxicity in HEK293 and SH-SY5Y cells at the tested concentrations of 5 and 10 µM.

## Figures and Tables

**Table 1:** Cytotoxicity of compounds **4a**–**4h** and the reference compound tBHQ in HEK293 cells.

Compound	Concentration	% Cell Viability
**4a**	10 µM	91.1 ± 2.6
	15 µM	95.0 ± 2.6
**4b**	10 µM	71.3 ± 3.2 *
	15 µM	32.2 ± 2.7 ***
**4c**	10 µM	73.1 ± 3.1 *
	15 µM	26.9 ± 2.7 ***
**4d**	10 µM	97.2 ± 2.5
	15 µM	91.0 ± 3.7
**4e**	10 µM	96.4 ± 3.6
	15 µM	86.3 ± 1.8 *
**4f**	10 µM	90.0 ± 0.7
	15 µM	41.5 ± 1.5 ***
**4g**	10 µM	100.4 ± 1.6
	15 µM	46.0 ± 3.7 ***
**4h**	10 µM	92.9 ± 3.5
	15 µM	92.3 ± 3.6
tBHQ	10 µM	87.8 ± 3.5

Means ± SEM of duplicates from at least four different cultures. * *p* ≤ 0.05 and *** *p* ≤ 0.001 as compared to the control cultures (one-way ANOVA with post hoc Dunnett’s test).

**Table 2:** Nrf2 induction potencies of compounds **4a**–**4h** and the reference compound tBHQ in Nrf2/ARE-luciferase reporter cells.

Compound	CD (µM)
tBHQ	0.6 ± 0.1
**4a**	1.0 ± 0.1
**4b**	0.4 ± 0.1
**4c**	0.3 ± 0.1
**4d**	0.7 ± 0.1
**4e**	1.0 ± 0.1
**4f**	0.4 ± 0.1
**4g**	0.7 ± 0.2
**4h**	0.5 ± 0.1

Data were plotted as concentration–response curves and fitted to a nonlinear equation. Data are expressed as the concentration required to double the specific luciferase reporter activity (CD) and are means ± SEM of at least 3 different experiments.

**Table 3:** Cytotoxicity of compounds **4a**–**4h** and the reference compound tBHQ in SH-SY5Y cells.

Compound	Concentration	% Cell Viability
**4a**	5 µM	96.2 ± 3.4
	10 µM	85.9 ± 2.4
**4b**	5 µM	89.3 ± 4.2
	10 µM	72.6 ± 2.8 ***
**4c**	5 µM	91.7 ± 3.2
	10 µM	64.3 ± 7.8 ***
**4d**	5 µM	96.9 ± 3.7
	10 µM	87.4 ± 0.7
**4e**	5 µM	98.7 ± 3.3
	10 µM	89.4 ± 3.1
**4f**	5 µM	95.4 ± 3.9
	10 µM	86.9 ± 3.6
**4g**	5 µM	89.0 ± 2.1
	10 µM	54.1 ± 6.0 ***
**4h**	5 µM	102.1 ± 3.1
	10 µM	95.7 ± 5.5
tBHQ	5 µM	93.6 ± 2.1
	10 µM	91.2 ± 4.6

Means ± SEM of triplicates from at least three different cultures. *** *p* < 0.001 as compared to the control cultures (one-way ANOVA with post hoc Dunnett’s test).

**Table 4:** Primer sequences used in qRT-PCR.

Target	Forward Primer (5′-3′)	Reverse Primer (3′-5′)
NQO1	CCTGCCATTCTGAAAGGCTGGT	GTGGTGATGGAAAGCACTGCCT
HO1	CCAGGCAGAGAATGCTGAGTTC	AAGACTGGGCTCTCCTTGTTGC
GCLC	GGAAGTGGATGTGGACACCAGA	GCTTGTAGTCAGGATGGTTTGCG
GCLM	TCTTGCCTCCTGCTGTGTGATG	TTGGAAACTTGCTTCAGAAAGCAG
GAPDH	AAGGTGAAGGTCGGAGTCAA	AATGAAGGGGTCATTGATGG
*β*-actin	CCTGGCACCCAGCACATT	GGGCCGGACTCGTCATAC

## Data Availability

Data are contained within the article and Supplementary Materials.

## Supplementary Materials

The following supporting information can be downloaded at: https://www.mdpi.com/article/10.3390/molecules30092048/s1.

## Abbreviations

The following abbreviations are used in this manuscript:

AChEI	Acetylcholinesterase inhibitor
AD	Alzheimer’s disease
ARE	Antioxidant response element
ATF4	Activating transcription factor 4
A*β*	*β*-amyloid
Cul3	Cullin3
DMEM-F12	Dulbecco’s MEM high-glucose
DMSO	Dimethyl sulfoxide
FBS	Fetal bovine serum
GCLC	Glutamate-cysteine ligase catalytic subunit
GCLM	Glutamate-cysteine ligase regulatory subunit
GST	Glutathione S-transferase
HO-1	Heme oxygenase-1
Keap1	Kelch-like ECH-associated protein 1 suppressor
MTDLs	Multi-target-directed ligands
NFT	Neurofibrillary tangle
NQO1	NAD(P)H:quinone dehydrogenase 1
Nrf2	Nuclear factor erythroid-2-related factor 2
ROS	Reactive oxygen species
RNS	Reactive nitrogen species
tBHQ	Tert-butylhydroquinone
