# Supplementary material for: Nrf2 Activation and Antioxidant Properties of Chromone-Containing MTDLs for Alzheimer’s Disease Treatment
Source: Molecules. 2025 May 4;30(9):2048. doi: 10.3390/molecules30092048 (PMC12074128; doi:10.3390/molecules30092048)
Supplement: Supplementary file 1 [file molecules-30-02048-s001.zip › molecules-3546230-supplementary.pdf]

SUPPORTING INFORMATION

Nrf2 Activation and Antioxidant Properties of Chromone-Containing MTDLs for Alzheimer's Disease Treatment

Alexey Simakov [1,2], Stecy Chhor [2], Lhassane Ismaili [1]* and Hélène Martin [2]*

Table of contents

Synthesis and characterization of compounds S2-S4

HPLC of compounds 4b and 4g..S5

Synthesis of compounds 4a-h

General procedure for the synthesis of the Passerini adducts **4a**-**h**: a solution of the corresponding aldehyde (1 equiv, 1.05 mmol), isocyanide (1.11 equiv, 1.17 mmol) and acetic acid (1.11 equiv, 1.17 mmol) in CH_2Cl_2 (5ml) is stirred for 24 hours at room temperature. The solvent is then evaporated and the crude purified by flash column chromatography using CH_2Cl_2/MeOH/NH_3 (95:4.05:0.05) to afford the desired product.

2-(((1-benzylpiperidin-4-yl)methyl)amino)-2-oxo-1-(4-oxo-4H-chromen-3-yl)ethyl acetate (**4a**), yield: 34%. ^{1}H NMR (300 MHz, $CDCl_3$) δ 8.23 (s, 1H), 8.19 (d, J = 8.0 Hz, 1H), 7.71 (t, J = 7.8 Hz, 1H), 7.54 – 7.39 (m, 2H), 7.37 – 7.29 (m, 5H), 7.07 (t, J = 5.2 Hz, 1H), 6.08 (s, 1H), 3.64 (s, 2H), 3.32 – 3.18 (m, 1H), 3.14 – 3.04 (m, 1H), 3.04 – 2.93 (m, 2H), 2.20 (s, 3H), 2.17 – 2.04 (m, 2H), 1.78 – 1.64 (m, 2H), 1.63 – 1.49 (m, 1H), 1.48 – 1.30 (m, 2H). ^{13}C NMR (75 MHz, $CDCl_3$) δ 176.47, 169.59, 167.60, 156.31, 155.46, 135.93, 134.36, 129.81, 128.37, 127.64, 125.82, 125.75, 123.77, 119.52, 118.36, 68.56, 62.25, 52.52, 44.84, 35.43, 28.84, 20.84. Anal. Calcd. For $C_{26}H_{28}N_2O_5$: C, 69.63; H, 6.29; N, 6.25. Found: C, 69.85; H, 6.33; N, 6.19.

2-(((1-benzylpiperidin-4-yl)methyl)amino)-1-(6-bromo-4-oxo-4H-chromen-3-yl)-2-oxoethyl acetate (**4b**), yield: 27%. ^{1}H NMR (400 MHz, $CDCl_3$) δ 8.32 (d, J = 2.3 Hz, 1H), 8.21 (s, 1H), 7.79 (dd, J = 8.9, 2.4 Hz, 1H), 7.40 (d, J = 8.9 Hz, 1H), 7.35 – 7.29 (m, 4H), 6.84 (bs, 1H), 6.03 (s, 1H), 3.54 (s, 2H), 3.30 – 3.18 (m, 1H), 3.14 – 3.01 (m, 1H), 2.99 – 2.82 (m, 2H), 2.20 (s, 3H), 2.08 – 1.92 (m, 2H), 1.75 – 1.62 (m, 3H), 1.42 – 1.26 (m, 2H). ^{13}C NMR (101 MHz, $CDCl_3$) δ 175.33, 169.66, 167.32, 155.73, 155.17, 137.47, 129.52, 128.57, 128.39, 127.34, 125.18, 120.43, 119.84, 119.38, 68.59, 63.20, 53.23, 45.19, 35.89, 29.66, 20.98. Anal. Calcd. For $C_{26}H_{27}BrN_2O_5$: C, 59.21; H, 5.16; N, 5.31. Found: C, 59.40; H, 5.21; N, 5.28.

2-(((1-benzylpiperidin-4-yl)methyl)amino)-1-(6-chloro-7-methyl-4-oxo-4H-chromen-3-yl)-2-oxoethyl acetate (**4c**), yield: 40%. ^{1}H NMR (300 MHz, $CDCl_3$) δ 8.17 (s, 1H), 8.14 (s, 1H), 7.39 (s, 1H), 7.35 – 7.27 (m, 5H), 6.87 (bs, 1H), 6.04 (s, 1H), 3.53 (s, 2H), 3.32 – 3.16 (m, 1H), 3.14 – 3.00 (m, 1H), 2.96 – 2.87 (m, 2H), 2.51 (s, 3H), 2.20 (s, 3H), 2.01 – 1.94 (m, 3H), 1.70 – 1.63 (m, 1H), 1.58 – 1.45 (m, 1H), 1.37 – 1.26 (m, 1H). ^{13}C NMR (75 MHz, $CDCl_3$) δ 175.44, 169.71, 167.53, 155.52, 154.65, 143.98, 136.58, 132.65, 129.80, 128.43, 127.59, 125.49,

122.84, 120.22, 119.46, 68.56, 62.66, 52.82, 45.05, 35.65, 29.20, 21.07, 21.00. Anal. Calcd. For $C_{27}H_{29}ClN_2O_5$: C, 65.25; H, 5.88; N, 5.64. Found: C, 54.98; H, 5.95; N, 5.60.

2-(((1-benzylpiperidin-4-yl)methyl)amino)-1-(6-ethyl-4-oxo-4H-chromen-3-yl)-2-oxoethyl acetate (**4d**), yield: 50%. ^{1}H NMR (400 MHz, $CDCl_3$) δ 8.20 (s, 1H), 8.00 (d, *J* = 2.0 Hz, 1H), 7.55 (dd, *J* = 8.6, 2.2 Hz, 1H), 7.42 (d, *J* = 8.6 Hz, 1H), 7.33 – 7.27 (m, 4H), 6.96 (t, *J* = 5.6 Hz, 1H), 6.09 (s, 1H), 3.52 (s, 2H), 3.30 – 3.16 (m, 1H), 3.13 – 3.01 (m, 1H), 2.96 – 2.83 (m, 2H), 2.77 (q, *J* = 7.6 Hz, 2H), 2.21 (s, 3H), 2.04 – 1.90 (m, 2H), 1.74 – 1.61 (m, 3H), 1.57 – 1.46 (m, 1H), 1.38 – 1.31 (m, 1H), 1.28 (t, *J* = 7.6 Hz, 3H). ^{13}C NMR (101 MHz, $CDCl_3$) δ 176.84, 169.73, 167.68, 155.21, 154.88, 142.28, 134.77, 129.48, 128.36, 123.96, 123.60, 119.38, 118.33, 68.62, 53.22, 45.18, 28.52, 20.99, 15.58. Anal. Calcd. For $C_{28}H_{32}N_2O_5$: C, 70.57; H, 6.77; N, 5.88. Found: C, 70.18; H, 5.69; N, 5.93.

2-((2-(1-benzylpiperidin-4-yl)ethyl)amino)-2-oxo-1-(4-oxo-4H-chromen-3-yl)ethyl acetate (**4e**), yield: 61%. ^{1}H NMR (300 MHz, $CDCl_3$) δ 8.27 – 8.12 (m, 2H), 7.81 – 7.67 (m, 1H), 7.56 – 7.41 (m, 2H), 7.40 – 7.30 (m, 5H), 6.87 (bs, 1H), 6.05 (s, 1H), 3.73 (s, 2H), 3.43 – 3.29 (m, 1H), 3.29 – 3.15 (m, 1H), 3.13 – 2.96 (m, 2H), 2.21 (s, 3H), 2.18 – 2.06 (m, 2H), 1.81 – 1.63 (m, 2H), 1.57 – 1.31 (m, 5H). ^{13}C NMR (75 MHz, $CDCl_3$) δ 176.58, 169.70, 167.51, 156.36, 155.53, 134.49, 130.31, 128.63, 128.25, 125.85, 125.81, 123.79, 119.52, 118.48, 68.54, 62.16, 52.96, 36.99, 35.57, 32.32, 30.64, 30.40, 20.96. Anal. Calcd. For $C_{27}H_{30}N_2O_5$: C, 70.11; H, 6.54; N, 6.06. Found: C, 70.45; H, 6.58; N, 6.03.

2-((2-(1-benzylpiperidin-4-yl)ethyl)amino)-1-(6-bromo-4-oxo-4H-chromen-3-yl)-2-oxoethyl acetate (**4f**), yield: 47%. ^{1}H NMR (300 MHz, MeOD) δ 8.37 (s, 1H), 8.25 (s, 1H), 7.94 (d, *J* = 8.9 Hz, 1H), 7.58 (d, *J* = 8.9 Hz, 1H), 7.49 – 7.44 (m, 5H), 6.02 (s, 1H), 4.20 (s, 2H), 3.68 – 3.60 (m, 1H), 3.48 – 3.34 (m, 3H), 2.97 – 2.75 (m, 2H), 2.15 (s, 3H), 2.04 – 1.89 (m, 2H), 1.69 – 1.30 (m, 5H). ^{13}C NMR (75 MHz, $CDCl_3$) δ 169.53, 167.26, 155.61, 155.47, 137.30, 129.46, 128.42, 128.3, 127.37, 125.02, 120.33, 119.71, 119.28, 68.43, 62.97, 53.06, 45.02, 35.78, 29.42, 20.86. Anal. Calcd. For $C_{27}H_{29}BrN_2O_5$: C, 59.90; H, 5.40; N, 5.17. Found: C, 60.19; H, 5.45; N, 5.21.

2-((2-(1-benzylpiperidin-4-yl)ethyl)amino)-1-(6-chloro-7-methyl-4-oxo-4H-chromen-3-yl)-2-oxoethyl acetate (**4g**), yield: 32%. ^{1}H NMR (400 MHz, $CDCl_3$) δ 8.17 (s, 1H), 8.15 (s, 1H), 7.39 (s, 1H), 7.36 – 7.28 (m, 4H), 6.74 (t, *J* = 5.3 Hz, 1H), 6.03 (s, 1H), 3.49 (s, 2H), 3.38 –

3.19 (m, 2H), 2.95 – 2.79 (m, 2H), 2.51 (s, 3H), 2.21 (s, 3H), 1.97 – 1.80 (m, 2H), 1.70 – 1.54 (m, 2H), 1.52 – 1.39 (m, 2H), 1.37 – 1.18 (m, 3H). ^{13}C NMR (101 MHz, $CDCl_3$) δ 175.48, 169.66, 167.29, 155.38, 154.69, 144.00, 132.67, 129.52, 128.34, 127.24, 125.57, 122.89, 120.24, 119.57, 68.49, 63.45, 53.78, 37.39, 35.88, 33.32, 31.97, 21.06, 20.97. Anal. Calcd. For $C_{28}H_{31}ClN_2O_5$: C, 65.81; H, 6.11; N, 5.48. Found: C, 66.12; H, 6.07; N, 5.51.

2-((2-(1-benzylpiperidin-4-yl)ethyl)amino)-1-(6-ethyl-4-oxo-4H-chromen-3-yl)-2-oxoethyl acetate (**4h**), yield: 47%. ^{1}H NMR (400 MHz, $CDCl_3$) δ 8.17 (s, 1H), 8.02 – 7.95 (m, 1H), 7.53 (dd, J = 8.6, 1.4 Hz, 1H), 7.40 (d, J = 8.6 Hz, 1H), 7.31 – 7.26 (m, 2H), 7.25 – 7.19 (m, 3H), 6.80 (t, J = 5.5 Hz, 1H), 6.05 (s, 1H), 3.48 (s, 2H), 3.33 – 3.16 (m, 2H), 2.91 – 2.80 (m, 2H), 2.74 (q, J = 7.6 Hz, 2H), 2.18 (s, 3H), 1.94 – 1.79 (m, 2H), 1.65 – 1.52 (m, 2H), 1.49 – 1.35 (m, 2H), 1.30 – 1.21 (m, 6H). ^{13}C NMR (101 MHz, $CDCl_3$) δ 176.82, 169.71, 167.56, 155.19, 154.87, 142.26, 134.78, 129.72, 128.37, 127.43, 123.93, 123.60, 119.41, 118.35, 68.52, 62.97, 53.41, 53.37, 37.30, 35.87, 33.12, 31.57, 28.51, 20.96, 15.57. Anal. Calcd. For $C_{29}H_{34}N_2O_5$: C, 71.00; H, 6.99; N, 5.71. Found: C, 69.72; H, 7.05; N, 5.68.

Purity HPLC (Method HPLC for P4b and P4g compounds)

Reagents:

- Solution of KH_2PO_4 at 0.1 gL^{-1} with H_3PO_4 at 0.5 gL^{-1}
- Methanol R

Equipment: HPLC/UV Chromaster HITACHI

Chromatographic condition:

- Mobile phase A: Solution of KH_2PO_4 at 0.1 gL^{-1} with H_3PO_4 at 0.5 gL^{-1}
- Mobile phase B: Methanol R
- Flow rate: 0.8 ml/min
- Wavelength: UV at 205 nm
- Column temperature: 40 °C
- Injection Volume: 20 µl
- Column: BDS Hypersil C18 – 250x4.6 mm – 5 µm
  Thermo Scientific – Ref 28105-254630
- HPLC mode: isocratic (phase A 70% - phase B 30 %)

Preparation of solution

- Sample solution at 10 mgL^{-1} in methanol

- HPLC chromatogram of 4b

Fichier: C:\Enterprise\Projects\UFC (L Ismaili)\Result\2025.03.31 - 2 pdts PA (03.2025)\PA02_002.dat
Méthode: C:\Enterprise\Projects\UFC (L Ismaili)\Method\LI_PA_(70-30_0.8_20').met

Volume: 20 µL   Vial # 1   Acquisition: 31/03/2025 15:02:26 (GMT +01:00)

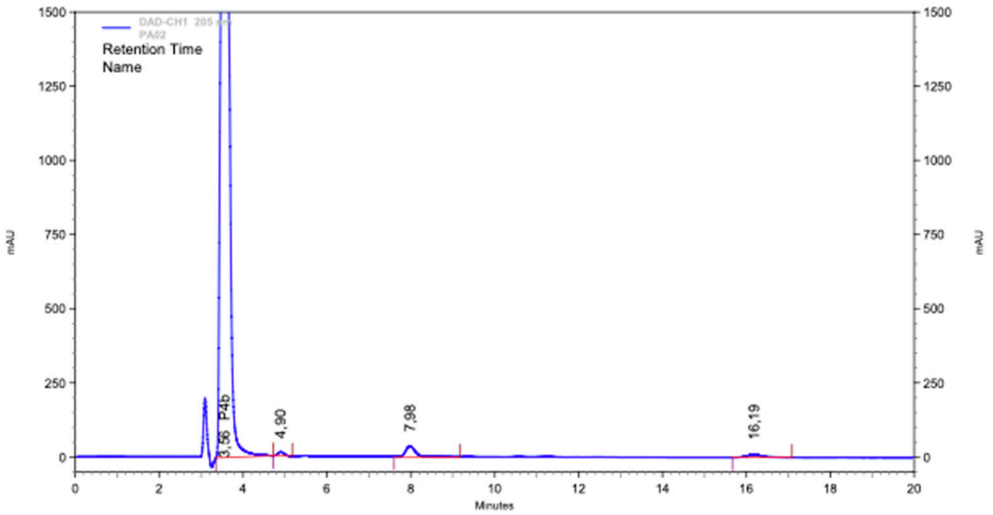

DAD-CH1 205 nm
Results

N°	Temps (min)	Nom	Surface	Area (%)
1	3,56	P4b	110713400	96,73
2	4,90		549338	0,48
3	7,98		2339888	2,04
4	16,19		848858	0,74

HPLC chromatogram of 4g

Fichier: C:\Enterprise\Projects\UFC (L Ismaili)\Result\2025.03.31 - 2 pdts PA (03.2025)\PA07_003.dat
Méthode: C:\Enterprise\Projects\UFC (L Ismaili)\Method\LI_PA_(70-30_0.8_20').met

Volume: 20 µL   Vial # 2   Acquisition: 31/03/2025 15:24:19 (GMT +01:00)

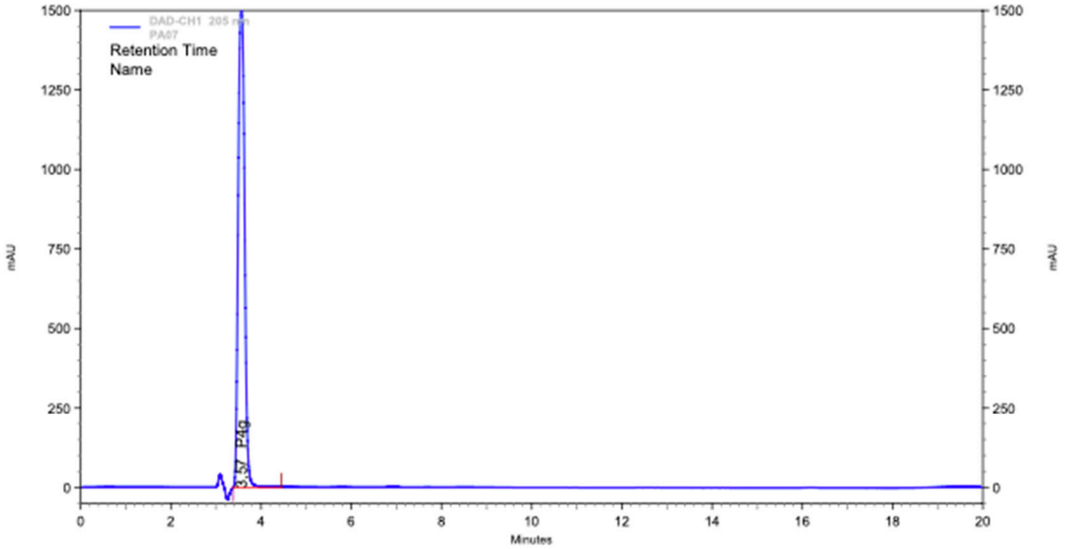

DAD-CH1 205 nm
Results

N°	Temps (min)	Nom	Surface	Area (%)
1	3,57	P4g	59400887	100,00
